# Supplementary material for: A prophylactic subcutaneous dose of the anticoagulant tinzaparin does not influence qPCR-based assessment of circulating levels of miRNA in humans
Source: PLoS One. 2022 Nov 3;17(11):e0277008. doi: 10.1371/journal.pone.0277008 (PMC9632808; doi:10.1371/journal.pone.0277008)
Supplement: Attachment — Submitted filename: Response to Reviewers.docx [file pone.0277008.s002.docx]

# Response to the reviewers

Reviewer #1: The authors of the manuscript entitled: “A prophylactic dose of Tinzaparin does not influence qPCR-based assessment of circulating levels of miRNA in humans” report the results of the study that aimed to investigate whether a prophylactic dose of the LMWH Tinzaparin influences qPCRbased quantification of circulating miRNAs in blood samples of patients enrolled previously into an ongoing study on acute compartment syndrome (BioFACTS, Clinicaltrials.gov, Identifier: NCT04674592).
In the group of 16 patients with traumatic tibial fractures on tinzaparin (vs. 16 of healthy controls no significant difference was observed for cDNA synthesis or qPCR when comparing tinzaparin treated patients with untreated controls. Among the tinzaparin treated patients, plasma levels of six endogenous miRNAs (hsa-let-7i-5p, hsa-miR-30e-5p, hsa-miR-222-3p, hsa-miR-1-3p, hsa-miR-133a-3p, hsa-miR-133b) were measured before and 1–6 hours after a subcutaneous injection of Tinzaparin 4500IU revealing no significant effect for any of the investigated miRNAs.

The topic is of interest, however, there are some relevant issues related to the manuscript.

In this small sample size study (groups 16 vs 16 patients) the large difference in the age of patients in the study and control group (Tinzaparin 48 (15-67) years, control 32 (18-50) appears problematic, the authors should have at least attempted to adjust their analyzes for some baseline confounders.
This is further exacerbated by the fact that the patients in the study group were sampled at different timepoints after the fracture.
How can the differences be attributed directly to the fact of tinzaparin therapy ? What could be the impact of comorbidities?

**Response: We thank the reviewer for this insightful comment. In our understanding of the current literature, the pharmacokinetics of tinzaparin are not affected by age or gender while severe renal impairment (GFR < 30 ml/min), may reduce clearance (1). I****n the BioFACTS study, renal impairment (GFR < 35 ml/min) along with other diseases affecting muscle tissue, are exclusion criteria and all patients in our control group were healthy. Therefore, we believe it is unlikely that differences in pharmacokinetics of tinzaparin would affect our analyses. Also, we compare miRNA levels between two time-points but within the same patient. This design should preclude any effect of age or gender differences between the groups. This is clarified under “Discussion”, page 15 row 265-272. Unfortunately, according to our statistical advisor, our sample size precludes comparisons stratified for several covariates. These limitations, along with the inherent limitations related to our small sample size, are now clarified under “Limitations”, page 15 row 281.**

The authors should further explain the rationale for selecting the described group of microRNA for analysis in their study.

**Response: We have added further clarifications to the selection of miRNAs in the amended version of the manuscript, page 10 row 170. This part of the methods section now reads:**

**“Six miRNAs were investigated based on previously observed effects of intravenous heparin treatment (2), of muscle specificity given the possibly of muscle injury following a tibial fracture (hsa-miR-1-3p, hsa-miR-133a-3p and hsa-miR-133b) (3) or suitability as stable circulating miRNAs (internal reference Qiagen); hsa-let-7i-5p, hsa-miR-30e-5p, hsa-miR-222-3p.”**

The methodology of sample and microRNA analysis appears appropriate and sufficiently described.

The authors state at the end of the discussion “Our results are important for clinical scientist conducting biomarker-focused research in clinical settings. As of today, many hospitalized patients receive venous thromboembolism prophylaxis, often with a similar regimen as in the current study. Moreover, the ongoing Sars-Cov2 pandemic has contributed to an increase in LMWH-treated patients, given the strong treatment recommendations for thromboembolism prophylaxis [19]. Our results provide guidance when miRNA analysis is performed in patients on Tinzaparin treatment.”
The authors should more clearly and specifically explain to the reader why their results are reportedly “important for clinical scientists”.

**Response: The last paragraph of the discussion is now rewritten to explain the importance of the result. “Our results should be reassuring for clinical scientist, indicating that miRNA levels seem unaffected by a prophylactic subcutaneous dose of Tinzaparin. Page 15, row 278-280.**

The authors should explicitly list the limitations of their study in the discussion section.

**Response: A “Limitations” heading in now added in the end of the discussion. Page 15, row, 281-291.**

A serious linguistic editing is required before the manuscript could be considered for further review and consideration for publication.

**Response: The amended version of the manuscript has been revised for language by ”Anchor English” (https://www.anchorenglish.com/).**

Reviewer #2: The topic of the study is interesting. It focuses on an important aspect that should be considered when miRNAs are studied from plasma.
However, I cannot understand some points.
First, UniSp2-4-5 have been added before RNA extraction to verify that Tinzaparin does not affect this step (as demonstrated in Fig.2). Then, UniSp6 and UniSp5 have been used to test cDNA synthesis step. Don’t UniSp2-4-5 already answer to this question? Indeed, since with the first step the authors conclude that Tinzaparin treatment does not affect RNA extraction also the following steps are included. Vice versa, cDNA synthesis step would be analyzed separately in the case of Tinzaparin treatment would affect the first result. To rescue the data about the cDNA synthesis, maybe, the data could be presented in a different order: first cDNA synthesis then RNA extraction.

**Response: We thank the reviewer for the opportunity to make this part of the result section clearer to the reader. The effects of tinzaparin could potentially influence both RNA extraction efficiency and cDNA synthesis and by only using UniSp2-4-5 spike-ins we would not be able to distinguish between these two steps. However, based on the recommendations provided by the reviewer, we have switched the order of the result segments so that the investigation on the effects on cDNA synthesis precedes the segment including the effect on RNA extraction. We hope this will make our methodology clearer.**

Finally, in fig. 4 miR-1-3p, miR-133a-3p and miR133b are characterized by a high standard deviation. A different graph for all the six miRNAs should be also presented. Particularly, the segment that joins the first point to the second point should be shown for each patient.

**Response: We thank the reviewer for bringing up a valuable point. We have remade the graphs so that they in addition to mean delta fold change values include individual data points and lines to join the pre and post values.**

1.	Hoy SM, Scott LJ, Plosker GL. Tinzaparin sodium: a review of its use in the prevention and treatment of deep vein thrombosis and pulmonary embolism, and in the prevention of clotting in the extracorporeal circuit during haemodialysis. Drugs. 2010;70(10):1319-47.

2.	Jes-Niels B. Heparin Selectively Affects the Quantification of MicroRNAs in Human Blood Samples. Clin Chem. 2013;59(7):1125-6.

3.	Horak M, Novak J, Bienertova-Vasku J. Muscle-specific microRNAs in skeletal muscle development. Dev Biol. 2016;410(1):1-13.
